# Challenges in cochlear implant care for patients with migration backgrounds: Evaluating (Hr)QoL

**DOI:** 10.1016/j.jmh.2026.100396

**Published:** 2026-01-14

**Authors:** Susann Thyson, Kai G. Kahl, Maika Werminghaus, Thomas Klenzner

**Affiliations:** a Cochlea Implant Center Duesseldorf, Department Of Otorhinolaryngology, Medical Faculty and University Hospital Duesseldorf, Heinrich-Heine-University Duesseldorf, Moorenstraße 5, 40225 Duesseldorf, Germany; b Department of Psychiatry, Social Psychiatry and Psychotherapy, Hannover Medical School, Carl-Neuberg-Str. 1, 30625 Hannover, Germany

**Keywords:** Human migration, Hearing disorders, Cochlea implantation, Gender, Hearing rehabilitation

## Abstract

**Objective:**

This study aims to assess and compare the quality of life (QoL) and health-related quality of life (HrQoL) of patients with cochlear implants (PwCI) and with or without a migration background (MB). It examines whether language proficiency and length of residence influence QoL and HrQoL outcomes in PwCI with a MB.

**Methods:**

Data from *n* = 82 PwCI were collected. QoL and HrQoL were measured using WHOQOL-BREF and Nijmegen Cochlear Implant Questionnaire (NCIQ), while CEFR assessed language proficiency.

**Results:**

PwCI with a MB had significantly lower WHOQOL-BREF global scores (Mdn = 62.50 / IQR = 25.00) than PmCI without a MB (Mdn = 75.00 / IQR = 25.00; *U* = 548.000, *Z* = -2.779, *p* = .005, *r* = 0.309). Similarly, NCIQ total scores were lower in PwCI with a MB (Mdn = 59.34, IQR = 21.53) than in controls (Mdn = 64.17, IQR = 19.98; *U* = 623.000, *Z* = –2.017, *p* = .044, *r* = 0.222). The language proficiency of PwCI with a MB in their second language, German, showed no correlation with the global score of the WHOQOL-BREF (Spearman's ρ = 0.240, *p* = .130). The language proficiency in German as a second language of PwCI with a MB shows a strong correlation with the total score on the NCIQ (Spearman's ρ = 0.428, *p* = .005), while length of residence showed no significant correlations.

**Discussion:**

PwCI with a MB tended to have lower QoL and HrQoL, potentially due to factors such as language barriers, and limited healthcare access. Language proficiency appeared to play a role, while length of residence showed no clear effect. Addressing linguistic and cultural barriers, could help improve healthcare access. Enhancing communication and support may facilitate greater participation in rehabilitation and treatment adherence, potentially leading to better QoL and more efficient use of healthcare resources.

## Highlights

• QoL and HrQoL were lower in cochlear implant patients with migration background.
• German language proficiency correlated with higher HrQoL scores, not with QoL scores.
• Male patients with migration background showed lowest global and environmental QoL.
• Length of residence showed no significant effect on QoL or HrQoL outcomes.
• Addressing language and cultural barriers may improve rehabilitation and healthcare use.

**Glossary**
CI: Cochlea Implant
CIVE: Certificated CI-provision institution (Cochlea-Implantat versorgende Einrichtung)
HrQoL: Health-related Quality of Life
MB: Migration Background
PwCI: Patients with Cochlea Implant
QoL: Quality of Life

## 1 Introduction

In Germany, >2.5 million people are estimated to have immigrated by 2022, marking the largest increase in the migrant population since 1950 (Federal Ministry of the Interior and Community and Federal Office for Migration and Refugees, 2024). According to the definition used by the German Federal Office for Migration and Refugees, a person is considered to have a migration background if they themselves, or at least one of their parents, did not acquire German citizenship at birth. This definition served as the basis for the classification applied in the present study.

The access to cochlear implantation is regulated within the framework of the German statutory health insurance, which covers the costs of diagnosis, surgery, and rehabilitation for individuals with medically confirmed severe to profound hearing loss. The German healthcare system provides universal and free coverage, and eligibility for cochlear implantation is based on audiological and medical criteria rather than citizenship or migration status. Consequently, both citizens and legally residing migrants have access to cochlear implant (CI) care through the same referral pathways. Typically, patients are referred to specialized tertiary CI care centers or university hospitals by otolaryngologists in ambulatory settings or hearing instrument specialists after conventional hearing aids have been deemed insufficient. At these CI care centers, comprehensive preoperative diagnostics including audiometry, speech perception tests, and radiological imaging are conducted to confirm candidacy. Following implantation, patients enter structured outpatient rehabilitation programs consisting of medical examinations, audiological adjustments and speech and language therapy (Lesinski-Schiedat et al., 2020).

The utilization of healthcare services is lower among people with a migration background (MB) than among non-migrants (Mead and Roland, 2009; World Health Organization, 2022). This disparity is often attributed to language barriers, cultural differences, limited knowledge of the healthcare system, or experiences of discrimination (World Health Organization, 2022; Kajikhina et al., 2023). Consequently, health issues may go undetected or be inadequately treated, which can negatively impact both quality of life (QoL) and health-related quality of life (HrQoL) for people with a MB. For instance, people with a MB often experience poorer mental health outcomes than their non-migrant peers (Lofvander et al., 2014; Toselli et al., 2014; Guo et al., 2019). Studies further indicate that differences exist in subjective well-being and QoL between men and women with a MB (Nissen et al., 2021; Regev and Slonim-Nevo, 2019). Men are more likely to report lower levels of well-being and reduced QoL (Nissen et al., 2021; Beiser and Hou, 2017).

### 1.1 QoL and HrQoL

Quality of life (QoL) includes various aspects that contribute to or influence an individual's overall well-being. These include personal and environmental factors, such as social, material, and economic components, as well as personal goals, expectations, cultural values, and physical and mental health (Karimi and Brazier, 2016; World Health Organization, 2024). General QoL incorporates elements indirectly related to health, including financial security and social relationships. Notably, QoL can be assessed in individuals who may not perceive themselves as affected by illness (Karimi and Brazier, 2016).

In contrast, Ojelabi et al. (2017) define HrQoL as a multidimensional construct encompassing disease symptoms, treatment side effects, overall health perception, and life satisfaction. Unlike general QoL measures, HrQoL assessments typically exclude non-health-related factors such as economic or material aspects (Karimi and Brazier, 2016).

### 1.2 QoL and HrQoL in patients with hearing disorders and cochlear implants (CI)

Hearing loss, particularly when acquired postlingually in adults, represents a profound sensory impairment with significant implications for HrQoL (Fellinger et al., 2007). Beyond the physical disability, hearing loss has extensive psychosocial consequences, including strained familial and social relationships, increased risk of social isolation, and a heightened prevalence of psychological distress such as depression and anxiety (Ciesla et al., 2016; Fellinger et al., 2007). These multifaceted challenges highlight the need for effective interventions to mitigate the detrimental effects of hearing loss on physical, psychological, and social well-being (Barbosa et al., 2015; Hogan et al., 2009).

The implantation of one or two CI provides individuals with severe, profound or total hearing loss the opportunity to regain speech comprehension (World Health Organization, 2021). According to Lailach et al. (2024) CI-treatment can improve HrQoL; however, its impact on overall QoL after the CI treatment appears to be minimal or negligible. Preliminary findings by Fan et al. (2024) indicate that patients with CI (PwCI) who also have a MB report lower HrQoL associated with their CI treatment compared to PwCI without a MB. This group appears to face intersectional challenges in terms of both QoL and HrQoL due to compounded difficulties, including limited access to health care and experiences of discrimination related to their MB (Ma and Joshi, 2022).

There is an increased need for research on the QoL and HrQoL of individuals with a MB and hearing disorders (Nesterko et al., 2013; van der Boor et al., 2020; Fan et al., 2024). In Germany, crucial information is lacking regarding chronic disease management, healthcare utilization, and access to rehabilitation services for this patient group (Integration Commissioner, 2024). Therefore, the present study aims to assess and compare the QoL and HrQoL of PwCI with a MB and those without a MB. In addition, the study aims to examine whether German language proficiency, duration of residence in Germany and gender influence QoL and HrQoL outcomes in PwCI with a MB.

Research Questions:

A) Do general QoL scores, as measured by the WHOQOL-BREF questionnaire (WHOQOL-BREF), differ between PwCI with a MB and PwCI without a MB?
B) Do HrQoL scores, as assessed by the Nijmegen Cochlear Implant Questionnaire (NCIQ), differ between PwCI with a MB and PwCI without a MB?
C) Is there a correlation between German language proficiency, as assessed by the Common European Framework of Reference for Languages (CEFR), length of residence in Germany, and the results of the WHOQOL-BREF and NCIQ?
D) To what extent do gender-specific differences emerge within the groups regarding QoL?

## 2 Methods

### 2.1 Study design and participants

The study employed a retrospective observational design based on self-administered questionnaires completed at a single time point.

In the specific setting of this study, which was conducted at a quality certificated CI-provision institution (Cochlea-Implantat versorgende Einrichtung, CIVE) (Stover et al., 2023) at a university hospital in western Germany, all PwCI had already received one or two cochlear implants and were enrolled in standard postoperative rehabilitation at the time of data collection. The centre serves a linguistically diverse patient population, mainly from Europe, and Asia, reflecting the demographic characteristics of the broader catchment area.

For the questionnaire survey, each PwCI was assessed at a single data collection time point. Participants were identified within routine clinical care at CIVE. Data collection was conducted during regularly scheduled outpatient rehabilitation appointments. Data were collected between March 2023 and September 2024. Participants were recruited through the Hearing Center of the Department of Otorhinolaryngology, University Hospital Düsseldorf. Participants were eligible for inclusion if they were adults (≥18 years), had been fitted with one or two CI for at least six months, had achieved stable CI fitting, and were attending routine outpatient rehabilitation at the CIVE during the data collection period. Data from participants who self-reported cognitive or psychiatric conditions that could interfere with questionnaire completion, such as dementia, schizophrenia, or depression, were excluded from the analysis.

The WHOQOL-BREF and the NCIQ were administered as part of the standard care during CI rehabilitation, and questionnaires were returned on the same day. The questionnaires were administered in a paper-and-pencil format. All patients completed the instruments independently without assistance. The questionnaires were distributed by the responsible speech-language therapist as part of the standard of care during routine rehabilitation. As per clinical protocol, all patients at the center complete these questionnaires once per year.

The CEFR was assessed in PwCI with a MB as part of a companion study during the same time period. Patient-reported outcomes were also obtained using paper-and-pencil format. All PwCI with a MB were literate in both their native language and German. In addition, participants provided information on the duration of their residence in Germany and their country of origin.

The study included data from PwCI with and without a MB. The control group consisted of monolingual, German-speaking adult (≥18 years) PwCI. All PwCI in the control group had also received one or two CIs for at least six months and had achieved stable CI fitting. Similarly, no participants with self-reported cognitive impairments such as dementia or schizophrenia, or with depression, were included in the control group.

Regarding sample selection, the two groups were matched by frequency with respect to age and gender to reduce imbalance between groups. No 1:1 individual matching was performed.

### 2.2 Questionnaires

#### 2.2.1 *WHOQOL-BREF*

The WHOQOL-BREF was used to evaluate PwCIs' QoL across four key domains: physical health, psychological well-being, social relationships, and environmental factors (World Health Organization, 1998). The WHOQOL-BREF is a widely used instrument for assessing QoL across diverse populations and cultural contexts. The questionnaire consists of 26 items, each scored on a five-point Likert scale, measuring intensity, capacity, or frequency of experiences, with higher scores indicating better QoL. Domain scores were calculated according to the WHO's standard scoring guidelines and subsequently transformed to a 0–100 scale for ease of interpretation. The German version of the WHOQOL-BREF has demonstrated acceptable to good internal consistency across all domains in a large validation sample (*n* = 2408), with Cronbach’s *α* values (referred to as *α* in the following) of *α* = 0.88 for the physical health domain, *α* = 0.83 for the psychological well-being domain, *α* = 0.76 for the social relationships domain, and *α* = 0.78 for the environmental factors domain (Skevington et al., 2004). The WHOQOL-BREF is available in several languages and was provided in participants’ native languages when necessary. Completion time was approximately 5–7.

#### 2.2.2 *NCIQ*

The NCIQ is a disease-specific questionnaire designed to assess the HrQoL of patients before and after cochlear implantation(Hinderink et al., 2000). The NCIQ was used to assess the HrQoL of all participants across six domains: Sound Perception Basic, Sound Perception Advanced, Speech Production, Self-steem, Activity, and Social Interactions. The NCIQ is a validated instrument designed to measure the impact of cochlear implantation on various QoL dimensions. It consists of 60 items, each scored on a five-point Likert scale, with higher scores indicating better outcomes. In the original validation study of the NCIQ, the instrument demonstrated good to excellent internal consistency across all domains. The total score achieved an *α* of 0.91. High internal reliability was also reported for all six subdomains, with *α* values of *α* = 0.90 for Sound Perception Basic, *α* = 0.87 for Sound Perception Advanced, *α* = 0.89 for Speech Production, *α* = 0.88 for Self-esteem, *α* = 0.87 for Activity, and *α* = 0.88 for Social Interaction (Plath et al., 2022). These values indicate strong internal consistency of the NCIQ across its subdomains. Completion time was approximately 10–15 min. The questionnaire was completed in the participants' second language.

#### 2.2.3 *CEFR*

To provide a comprehensive sample description and enable correlation analyses, PwCI with a MB completed a self-assessment of their proficiency in their second language, German, using the CEFR. The CEFR is an internationally recognized framework for describing and evaluating language proficiency across different linguistic competencies. It categorizes proficiency into six levels: A1, A2, B1, B2, C1, and C2 (Council of Europe, 2001). These levels progress from basic to proficient users and enable systematic measurement of language competencies across a wide spectrum of abilities. The CEFR enables self-assessment of various language components, including listening, reading, spoken interaction, spoken production and writing. The CEFR was made available to PwCI with a MB in their first language, if required. Participants assigned one of the six levels to each language component. A mean score was calculated based on these self-assessments. Completion time was approximately 2–4 min.

### 2.3 Data analysis

All statistical analyses were performed using SPSS (Version 30) and Microsoft Excel. The level of statistical significance was set at *p* < .05 for all analyses unless otherwise specified.

To determine whether parametric assumptions were met, the distribution of all continuous variables was assessed using the Shapiro-Wilk test. As most questionnaire scores were not normally distributed, non-parametric tests were applied. Group differences between patients with and without a migration background were examined using the Mann-Whitney U test. Effect sizes for these comparisons were calculated using Pearson’s r, derived from the z-values of the test statistics. Associations between continuous variables (e.g., WHOQOL-BREF global score, NCIQ total score, language proficiency, and length of residence) were analysed using Spearman’s rank-order correlation coefficients. To explore gender-specific differences, comparative analyses across the four gender subgroups (female with MB, female without MB, male with MB, male without MB) were conducted using the Kruskal-Wallis test. Where the overall test indicated significant group differences, post-hoc pairwise comparisons were performed with Bonferroni correction. Internal consistency of the questionnaire was assessed using Cronbach’s *α*. Cronbach’s *α* is a commonly used statistic to evaluate the degree of agreement among multiple items within a questionnaire. Values of *α* ≥ 0.70 are generally considered to indicate acceptable to high internal consistency of the instrument (Cronbach, 1951). In addition, multiple linear regression analyses were conducted to examine whether migration background remained a significant predictor of global WHOQOL-BREF and NCIQ scores after controlling for relevant covariates. Regression models included age, gender, type of CI, hearing mode, duration of residence, and duration of CI use as predictors. Regression assumptions (linearity, independence, homoscedasticity, multicollinearity, and influential outliers) were evaluated using standard diagnostic procedures such as residual plots, Durbin–Watson statistics, variance inflation factors, and Cook’s distance.

## 3 Results

### 3.1 Participants

In total, data were collected from *n* = 82 participants. Of these, 41 PwCI with a MB were assessed. As shown in Table 1, 20 of the PwCI with MB were female and 21 were male, with a mean age of 57 years (SD = 16) at the time of testing. They had been fitted with one or two CIs for a mean duration of 67 months (SD = 67).

**Table 1:** Hearing-related patient information.

	Group	
	PwCI and MB	PwCI without MB
	*n* = 41	*n* = 41
Gender ♀ | ♂	20 | 21	22 | 19
Age at testing (years) *M* ± SD	57 ± 16	58 ± 18
Duration of CI use (month) *M* ± SD	67 ± 67	79 ± 50
CI-System (%)		
Cochlear	63	51
MED-EL	27	39
Advanced Bionics	10	10
Hearing mode (%)		
Bilateral CI	20	22
Bimodal (CI + HA)	63	56
Single-Sided Deafness	17	22
Audiometric data		
PTA4*M ± SD	33.38 ± 7.71	31.68 ± 8.31
Speech comprehension⁎⁎ 65 dB *M* ± SD	46.83 ± 24.47	59.63 ± 24.92
Speech comprehension⁎⁎ 80 dB *M* ± SD	57.56 ± 25.35	66.46 ± 26.94

⁎ Measured for the CI (500 Hz, 1000 Hz, 2000 Hz, 4000 Hz). For bilateral CI participants, the last implanted CI was measured.

⁎⁎ Measured with Freiburger monosyllabic speech test. For bilateral CI participants, the last implanted CI was measured as well.

**Fig. 1:**
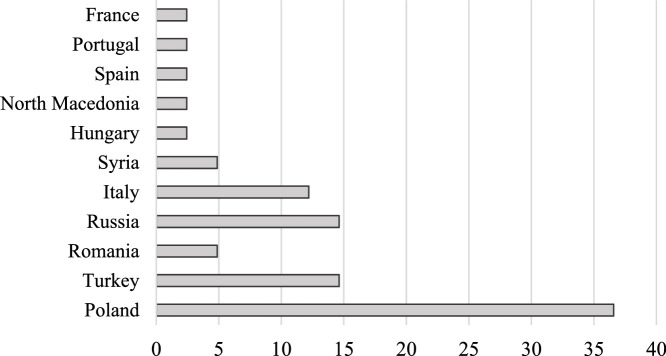
Countries of origin of the participants with migration backgrounds.

As shown in Fig. 1, the distribution of participants' countries of origin was heterogeneous, with individuals from 11 different countries included in the study. Fifteen participants were born in Poland, six each in Russia and Turkey, five in Italy, two each in Romania and Syria, and one each from Hungary, North Macedonia, Spain, Portugal, and France.

Furthermore, 68.29 % of the PwCI with a MB had lived in Germany for >30 years, 14.63 % for >20 years, 9.76 % for >10 years, and 7.32 % for >5 years.

The control group consisted of 41 monolingual German-speaking PwCI without a MB, of whom 22 were female and 19 were male. As shown in Table 1, participants had a mean age of 58 years (SD = 18) at the time of testing and had been fitted with one or two CIs for a mean duration of 79 months (SD = 50).

### 3.2 Questionnaires

#### 3.2.1 *WHOQOL-BREF*

In the present study, the WHOQOL-BREF demonstrated good internal reliability across all domains. Cronbach’s *α* for the global score was *α* = 0.85. Internal consistency for the domains was similarly high, with *α* = 0.85 for physical health, *α* = 0.83 for psychological health, *α* = 0.88 for social relationships, and *α* = 0.84 for the environment domain. These values indicate that the instrument showed excellent internal consistency within this study sample.

The data for the PwCI with a MB group were found to be not normally distributed according to the Shapiro-Wilk test (*p* = .004). Similarly, the data for the PwCI without a MB (control group) were also not normally distributed (*p* = .002). The WHOQOL-BREF global scores for PwCI with a MB were, on average, lower (Mdn = 62.50 / IQR = 25.00) than those of the control group (Mdn = 75.00 / IQR = 25.00). The results of a Mann-Whitney U test indicated that PwCI with a MB had significantly lower global scores on the WHOQOL-BREF compared to the control group (*U* = 548.000, *Z* = -2.779, *p* = .005). The effect size *r*, as measured by Pearson correlation coefficient, was *r* = 0.309, indicating a moderate effect.

**Fig. 2:**
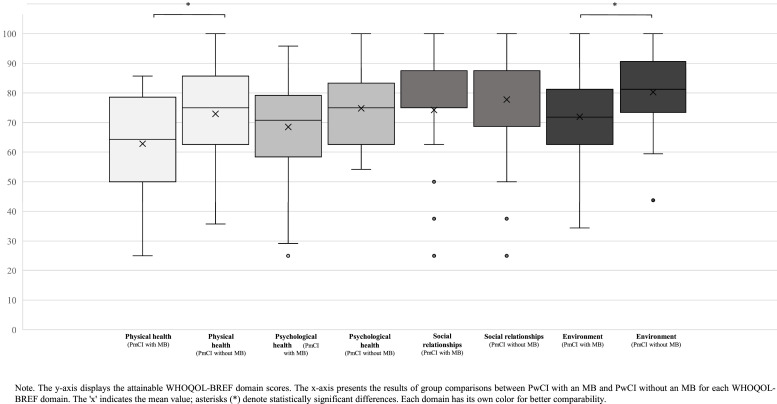
Group comparisons of WHOQOL-BREF domain scores between patients with cochlear implant (PwCI) with and without a migration background (MB).

A Mann–Whitney U test was also conducted to examine differences in WHOQOL-BREF domain scores for PwCI with a MB and PwCI without a MB. As shown in Fig. 2, the WHOQOL-BREF domain scores for physical health (*U* = 570.500, *Z* = –2.510, *p* = .012) and environment (*U* = 528.500, *Z* = –2.900, *p* = .004) revealed significant differences between PmCI with a MB and those without a MB. The effect size *r* for physical health was *r* = 0.277, indicating a weak effect, while the effect size *r* for environment was *r* = 0.320, indicating a moderate effect. By contrast, the WHOQOL-BREF domain scores for psychological health (*U* = 686.000, *Z* = –1.438, *p* = .150) and social relationships (*U* = 750.500, *Z* = –0.858, *p* = .391) showed no significant differences between the groups.

A multiple linear regression analysis was conducted to examine whether gender, duration of residence in Germany, duration of CI use, age, and hearing mode predicted global WHOQOL-BREF score. The overall regression model was not statistically significant, *F*(5, 35) = 0.17, *p* = .97, indicating that the combined predictors did not explain a significant proportion of the variance in global WHOQoL-BREF scores. The model accounted for only 2.3 % of the variance, *R²* = 0.023, and the adjusted *R²* was negative (-0.117), suggesting limited explanatory value. None of the individual predictors reached statistical significance. Gender (*t* = -0.08, *p* = .94), duration of residence in Germany (*t* = -0.42, *p* = .68), duration of CI use (*t* = 0.51, *p* = .61), age (*t* = 0.53, *p* = .60), and hearing mode (*t* = 0.34, *p* = .74) showed no significant associations with global WHOQOL-BREF scores.

Collinearity diagnostics indicated that multicollinearity was not a concern, with condition indices below the critical threshold and acceptable variance proportions across all predictors. Examination of residual statistics suggested that model assumptions were met; no influential outliers were detected based on Cook’s distance, Mahalanobis distance, or leverage values.

#### 3.2.2 *NCIQ*

In this study, the NCIQ showed consistently high internal reliability across the total score and all subdomains. The global score yielded a Cronbach’s *α* of *α* = 0.89. Reliability coefficients for the individual subdomains were similarly high, with *α* = 0.92 for Sound Perception Basic, *α* = 0.91 for Sound Perception Advanced, *α* = 0.93 for Speech Production, *α* = 0.91 for Self-esteem, *α* = 0.91 for Activity, and *α* = 0.91 for Social Interaction. Overall, these values demonstrate that the NCIQ performed with excellent internal consistency in the present sample.

The data for PwCI with a MB were normally distributed according to the Shapiro–Wilk test (*p* = .849). In contrast, the scores for PwCI without a MB were not normally distributed (*p* = .014). Due to the non-normal distribution in the group of PwCI without a MB and the small sample size, the non-parametric Mann–Whitney U test was chosen to examine group differences. The NCIQ global score for PwCI with a MB were, on average, lower (Mdn = 59.34, IQR = 21.53) than those of the control group (Mdn = 64.17, IQR = 19.98). The Mann–Whitney U test revealed a significant difference between the two groups, *U* = 623.000, *Z* = –2.017, *p* = .044. The effect size *r*, measured by Pearson’s correlation coefficient, was *r* = 0.222, indicating a weak effect.

**Fig. 3:**
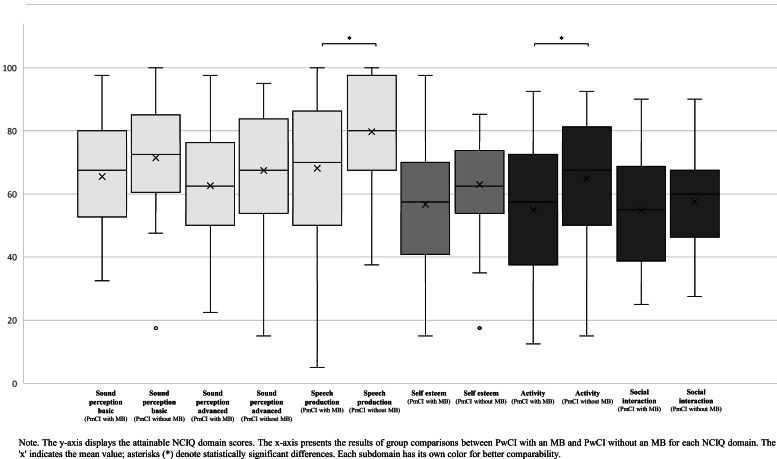
Group comparisons of NCIQ domain scores (percentages) between patients with cochlear implant (PwCI) with and without a migration background (MB).

A Mann–Whitney U test was also conducted to examine differences in NCIQ subdomain scores between PwCI with a MB and those without a MB. As shown in Fig. 3, significant group differences were observed for the subdomains Speech Production (*U* = 585.500, *Z* = -2.371, *p* = .018) and Activity (*U* = 599.500, *Z* = -2.239, *p* = .025). The corresponding effect sizes *r* were *r* = 0.261 for speech production and *r* = 0.247 for activity, both indicating a weak effect. By contrast, the subdomains Sound Perception Basic (*U* = 683.500, *Z* = -1.458, *p* = .161), Sound Perception Advanced (*U* = 715.500, *Z* = -1.161, *p* = .246), Self-esteem (*U* = 647.000, *Z* = -1.797, *p* = .072), and Social Interaction (*U* = 768.000, *Z* = -0.673, *p* = .501) showed no significant differences between groups.

Spearman’s rank-order correlation revealed a significant association between global NCIQ score and global WHOQOL-BREF score (*ρ*(82) = 0.383, *p* < .001). Positive correlations were also found between global NCIQ score and the WHOQOL-BREF domains physical health (*ρ*(82) = 0.368, *p* < .001), psychological health (*ρ*(82) = 0.364, *p* < .001), environment (ρ(82) = 0.404, *p* < .001) and social relationships (*ρ*(82) = 0.258, *p* = .019).

A multiple linear regression analysis was conducted to examine whether gender, duration of residence in Germany, duration of CI use, age, and hearing mode predicted global NCIQ scores. The overall regression model did not reach statistical significance, *F*(5, 35) = 1.75, *p* = .148, indicating that the combined predictors did not significantly explain variance in NCIQ global scores. The model accounted for 20 % of the variance, *R²* = 0.20, with an adjusted *R²* of 0.086. With regard to the individual predictors, none reached conventional levels of statistical significance with the exception of duration of CI use (*B* = 0.080, *t* = 2.10, *p* = .043), which showed a small but statistically significant positive association with global NCIQ scores. Gender (*t* = -0.53, *p* = .598), duration of residence in Germany (*t* = 1.25, *p* = .219), age (*t* = -1.86, *p* = .071), and hearing mode (*t* = 1.55, *p* = .131) did not show significant effects. Although age demonstrated a trend-level association (*p* = .071), it did not meet the threshold for statistical significance. Collinearity diagnostics indicated no violations of multicollinearity assumptions, with variance inflation factors ranging from 1.236 to 2.752 and condition indices remaining below critical thresholds. Residual statistics suggested that assumptions of linearity, homoscedasticity, and independence were adequately met. No influential outliers were detected based on Cook’s distance, Mahalanobis distance, or leverage values.

Although CI duration emerged as a statistically significant predictor, the effect size was small, indicating only a modest increase in NCIQ global scores with longer implant use. This suggests that subjective benefits captured by the NCIQ may continue to develop gradually over time; however, the clinical relevance of this effect is likely limited.

#### 3.2.3 *CEFR*

**Fig. 4:**
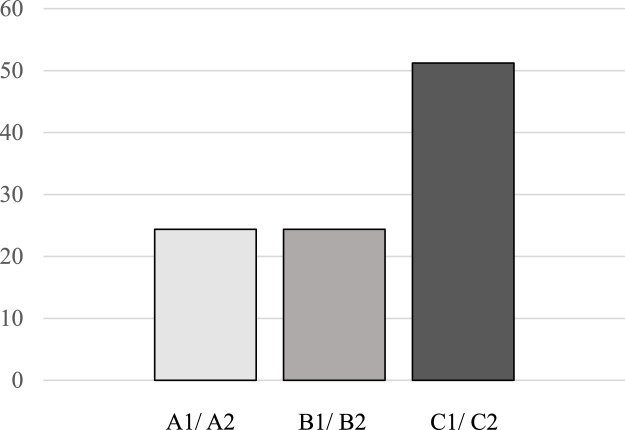
Language proficiency in German of the patients with cochlear implant and a migration background.

The PwCI with a MB assessed their proficiency in German as a second language using the CEFR. As shown in Fig. 4, on average, 24.39 % of the PwCI with a MB rated their language skills as elementary, while 75.61 % rated their overall German proficiency as either independent or proficient.

The language proficiency of PwCI with a MB in their second language, German, showed no correlation with the global score of the WHOQOL-BREF (Spearman's ρ = 0.240, *p* = .130).

The language proficiency in German as a second language of PwCI with a MB shows a strong correlation with the total score on the NCIQ (Spearman's ρ = 0.428, *p* = .005).

The length of residence in Germany among PwCI with a MB shows no correlation with the global score of the WHOQOL-BREF (Spearman's ρ = -0.165, *p* = .302). Similarly, there is no correlation between length of residence and global score on the NCIQ (Spearman's ρ = -0.212, *p* = .182).

#### 3.2.4 *Gender-specific differences*

A Kruskal-Wallis test revealed a statistically significant difference in global WHOQOL-BREF scores between the four gender groups: female PmCI with a MB and without a MB und male PmCI with a MB and without a MB (H(3) = 9.69, *p* = .021). Significant differences were also found for the physical health domain (H(3) = 8.05, *p* = .045) and the environmental domain (H(3) = 12.04, *p* = .007), but not for psychological heath (H(3) = 9.69, *p* = .551) or social relationships (H(3) = 9.69, *p* = .708). A Mann-Whitney U test revealed that male PmCI with a MB had significantly lower global WHOQOL-BREF scores (Mdn = 62.50) than female PmCI without a MB (Mdn = 75.00), *U* = 136.50, *Z* = –2.723, *p* = .006, *r* = 0.406. A second Mann-Whitney U test indicated a significant difference in the environmental WHOQOL-BREF domain, with male PmCI with a MB (Mdn = 60.00) reporting lower scores than female PmCI without a MB (Mdn = 75.00), *U* = 108.50, *Z* = –3.299, *p* < .001, *r* = 0.492. Both differences remained statistically significant after Bonferroni correction (adjusted *α* = 0.0083). All other pairwise comparisons between gender and mental burden subgroups yielded non-significant results after correction.

## 4 Discussion

The results provide important insights into the differences in QoL and HrQoL between PwCI with a MB and PwCI without a MB. The significantly lower WHOQOL-BREF scores observed in PwCI with a MB compared to the control group highlight the intersectional burden faced by this population. Intersectional burdens emerge when multiple disadvantages, such as social, health, or economic challenges, intersect and compound each other (Rai et al., 2020). For PwCI with a MB, this may indicate that, in addition to the challenges of living with one or two CIs, they may experience additional stressors such as chronic illness, psychological distress, social isolation, financial difficulties, or discrimination (Ma and Joshi, 2022). These factors can interact and reinforce each other, thus significantly impairing the QoL and well-being of the PwCI with a MB. The lower WHOQOL-BREF scores among PwCI with a MB suggest that their subjective QoL is not solely affected by hearing impairment but is also negatively influenced by additional factors such as socioeconomic status, disability, language barriers, and discrimination. Ma and Joshi (2022), along with Alvi and Zaidi (2017), emphasize the importance of considering the interplay between migration status, socioeconomic factors, and health to better understand health disparities. Reducing language barriers and providing access to health information within healthcare settings are critical interventions that can help address these disparities. In the context of CI care, it appears beneficial to offer questionnaires that take patients' native languages into account. In addition, assessing patients' subjective language proficiency can help to ensure that information is effectively communicated and understood, allowing for comprehensive care. Moreover, comparisons between groups with regard to gender indicate that gender-specific differences play a central role in the well-being and QoL of PmCI with a MB. In line with previous studies reporting lower well-being and reduced QoL among men with a MB, we found that male PmCI with a MB had significantly lower global and environment-related WHOQOL-BREF scores than female PmCI without a MB. This suggests that male migrants with hearing impairment may be particularly vulnerable to QoL limitations. A possible explanation is that men may benefit less from protective social resources or experience greater difficulties in adapting to the social and cultural environment of the host country (Beiser and Hou, 2017; Regev and Slonim-Nevo, 2019; Nissen et al., 2021). These gender-specific patterns highlight the importance of tailored interventions and support services that address the differing needs of men and women in order to promote health equity.

The significantly lower NCIQ scores among PwCl with a MB, similar to the findings of Fan et al. (2024), suggest that PwCI with a MB are more likely to experience health-related difficulties or face more severe health challenges than PwCI without a MB. One explanation for this might be that PwCI with a MB face obstacles related to language barriers, limited access to information, or restricted access to the healthcare system in general (Alvi and Zaidi, 2017; Kajikhina et al., 2023), which could affect their ability to fully understand and address aspects of their CI treatment and fitting. Furthermore, their rehabilitation process may be affected by inadequate follow-up care or less accessible auditory training. As a result, PwCI with a MB may derive less benefit from CI treatment than their non-migrant peers.

The distribution of countries of origin among PwCI with MB does not align with the assumptions of the healthy-migrant hypothesis, which suggests that migrants often display better health outcomes than the native-born population due to positive self-selection processes. In the present cohort, many individuals originated from regions where access to specialized hearing healthcare is limited, and migration frequently occurred under constrained socioeconomic or geopolitical conditions rather than as a voluntary, health-selective process. Moreover, candidates for cochlear implantation, by definition, present with severe to profound hearing loss, which inherently contradicts the premise of a health advantage at the point of migration. These contextual factors may help explain why PwCI with MB in our study reported lower QoL and HrQoL scores, indicating that the mechanisms proposed by the healthy-migrant hypothesis are not applicable in this specific clinical population (Abraido-Lanza et al., 1999; Rubalcava et al., 2008; Salas-Wright et al., 2018).

A strong correlation between German language proficiency (CEFR scores) and HrQoL outcomes underscores the crucial role of language proficiency in the rehabilitation and social integration of PwCI with a MB within the healthcare system. This finding is consistent with the work of Guo et al. (2019), who demonstrated that language barriers are significant predictors of QoL. However, this may be due to the fact that higher language proficiency facilitates communication with healthcare professionals, improves access to services, enhances social participation, and strengthens patients’ sense of autonomy, all of which are crucial determinants of HrQoL.

The duration of residence in Germany shows no significant correlation with QoL or HrQoL. This partially contradicts the assumption that a longer residence period automatically leads to better integration and improved QoL (Nesterko et al., 2013). This finding may suggest that other factors, such as experiences of discrimination or a lack of cultural sensitivity within healthcare services, play a more influential role in determining these outcomes.

### 4.1 Clinical implications

The findings suggest that more attention needs to be paid to the linguistic and cultural needs of PwCI with a MB. This could be achieved through measures such as employing multilingual professionals, providing translation services, and adapting rehabilitation programs to account for cultural differences. Since 2025, several of these measures have been implemented in the clinical unit where the study was conducted. The rehabilitation team now includes multilingual professionals who speak a first language other than German, and a video-based interpreting service with both live and ad-hoc interpreter availability is routinely employed to facilitate communication during diagnostics and therapy. Furthermore, patient information materials have been revised to include more visual elements and culturally sensitive adaptations, with the aim of improving accessibility and comprehension for patients with diverse linguistic backgrounds. In addition, integrative approaches, such as incorporating the International Classification of Functioning, Disability, and Health (ICF), may facilitate a more comprehensive assessment and support framework for patients.

### 4.2 Limitations and future research

The present study has limitations. One limitation is that language barriers may have influenced the results of the HrQoL assessment, as the NCIQ questionnaire was not available in the native language of PwCI with a MB. With regard to the language proficiency of PwCI with a MB, it was only measured through self-assessment, which may introduce subjective bias.

Although access to cochlear implantation in Germany is universally covered by statutory health insurance and does not depend on citizenship or migration status, structural barriers may still influence which individuals ultimately enter the rehabilitation system. Language barriers, limited health literacy, and a lack of reliable national data on healthcare utilization among people with a migration background may affect the representativeness of the study sample. As such, while access-related selection bias is likely lower than in healthcare systems with restricted insurance coverage, the findings may still underrepresent individuals with limited engagement in healthcare services.

In addition, the comparatively small sample size limits the statistical power of the analyses and may have reduced the ability to detect subtle but clinically relevant differences between groups. This is particularly important for domains in which patients with a migration background consistently showed lower scores, yet these differences did not reach statistical significance. It therefore remains possible that some effects were underestimated due to insufficient power rather than the absence of true group differences.

Future studies should include more objective measures and longitudinal designs to explore the causal relationships between migration, language proficiency, QoL and HrQoL. It is also important to recognize that the experiences and living conditions of people with a MB can vary widely, and not all members of this group face the same challenges. Addressing this heterogeneity in future research will provide a more nuanced understanding of the factors that influence health outcomes in this population.

## 5 Conclusion

The study results indicate that PwCI with a MB have significantly lower QoL and HrQoL scores compared to PwCI without a MB. These differences can be attributed to intersectional burdens resulting from the intersection of factors such as social isolation, language barriers, discrimination, and limited access to health information. In particular, language proficiency emerged as an influential factor affecting both QoL and HrQoL, as it facilitates access to relevant healthcare resources and enables active participation in rehabilitation programs. However, the study found that the length of time spent in the host country had no significant effect on QoL or HrQoL, suggesting that time alone is not sufficient to overcome social and health inequalities. The results highlight the urgent need to address linguistic and cultural barriers in healthcare to sustainably improve the QoL and HrQoL of this patient group.

Practical measures such as the use of translation services, the employment of multilingual professionals, and culturally sensitive rehabilitation programs could help to ensure inclusive and needs-based care for PwCI with a MB. Improved communication and support to address individual needs may lead to greater participation in healthcare and enhance adherence to treatment recommendations for patients with a MB. As a result, health problems may be reduced and healthcare resources may be used more efficiently. Strengthening the relationship between patients and the healthcare system can ultimately lead to positive long-term outcomes, both in terms of improved QoL and increased economic efficiency of healthcare services.

## Data availability

The research data underlying this study consist of sensitive patient data and therefore cannot be made publicly available due to data protection and privacy regulations. However, the dataset may be shared upon reasonable request to the corresponding author, provided that the request is scientifically justified and complies with ethical and legal requirements.

## Funding sources

This research did not receive any specific grant from funding agencies in the public, commercial, or not-for-profit sectors.

## CRediT authorship contribution statement

**Susann Thyson:** Writing – review & editing, Writing – original draft, Visualization, Validation, Supervision, Resources, Project administration, Methodology, Investigation, Formal analysis, Data curation, Conceptualization. **Kai G. Kahl:** Writing – review & editing, Validation, Supervision, Methodology, Formal analysis, Conceptualization. **Maika Werminghaus:** Writing – review & editing. **Thomas Klenzner:** Writing – review & editing, Validation, Supervision, Methodology, Formal analysis, Conceptualization.

## Declaration of competing interest

None.
